# Macrophages Do Not Express the Phagocytic Receptor BAI1/*ADGRB1*

**DOI:** 10.3389/fimmu.2019.00962

**Published:** 2019-05-03

**Authors:** Cheng-Chih Hsiao, Marlijn van der Poel, Tjakko J. van Ham, Jörg Hamann

**Affiliations:** ^1^Department of Experimental Immunology, Amsterdam Infection and Immunity Institute, Amsterdam UMC, University of Amsterdam, Amsterdam, Netherlands; ^2^Department of Neuroimmunology, Netherlands Institute for Neuroscience, Amsterdam, Netherlands; ^3^Department of Clinical Genetics, Erasmus University Medical Center Rotterdam, Rotterdam, Netherlands

**Keywords:** adhesion GPCRs, brain-specific angiogenesis inhibitors, macrophages, microglia, monocytes, phagocytic receptors

The highly organized life of metazoa requires the ability to remove cells that lose their function during embryonic and postnatal development or as part of routine tissue homeostasis (1, 2). Normally, these cells undergo programmed, apoptotic cell death, followed by their recognition, engulfment, and, finally, elimination through adjacent tissue cells and/or professional phagocytes. As preeminent phagocytic cells, resident macrophages and circulating monocytes are equipped with an arsenal of receptors that recognize the “eat-me” signals exposed by apoptotic corpses. These phagocytic receptors comprise scavenger receptors, immunoglobulin-containing proteins, and tyrosine kinases (1).

In a Nature paper in 2007, Park et al. described brain-specific angiogenesis inhibitor 1 (BAI1/*ADGRB1*) as a novel phagocytic receptor on macrophages (3). BAI1 is a member of the adhesion family of G protein-coupled receptors (GPCRs), which in humans comprises 33 non-canonical seven-span transmembrane receptors (4). Adhesion GPCRs possess large N-termini with various protein folds, equipped for (matri)cellular interactions, and a GPCR autoproteolysis-inducing (GAIN) domain that connects the extracellular part of the receptor to the seven-transmembrane region. A juxtamembranous GPCR-proteolysis site (GPS) within the GAIN domain facilitates autocatalytic cleavage of the majority of adhesion GPCRs into two fragments, which remain attached at the cell surface (5). Adhesion GPCRs are found in almost every cell type and adjust modalities in many organ systems. Based on their expression and function, adhesion GPCRs of subfamily E (EMR1/*ADGRE1*, EMR2/*ADGRE2*, EMR3/*ADGRE3*, EMR4/*ADGRE4*, and CD97/*ADGRE5*) and subfamily G (GPR56/*ADGRG1*, GPR97/*ADRGRG3*, and GPR114/*ADGRG5*) have been linked to the immune system (6, 7). BAI1 belongs to the subfamily B and is abundantly expressed in the brain, where it inhibits angiogenesis and, as recently reported, supports neurogenesis and synaptogenesis (8). The work by Park et al. and others established an additional function of BAI1 in apoptotic cell engulfment by macrophages and their brain equivalent, microglia (3, 9, 10). Through its N-terminal thrombospondin repeats, BAI1 binds phosphatidylserine, resulting in recruitment of ELMO1 and Dock180 to the C-terminus of the receptor, which function as guanine-exchange factors for Rac1 and thereby promote engulfment of apoptotic cells. Moreover, expression of BAI1 in primary human monocytes/macrophages and the mouse macrophage cell lines J774 and RAW264.7 was reported (3).

Ingestion of microbes, such as bacteria and fungi, is another phagocytic process executed by macrophages. A subsequent paper in 2011 described the ability of BAI1 to bind and engulf Gram-negative bacteria (11). Interaction of the thrombospondin repeats with bacterial membrane lipopolysaccharide triggered *Salmonella* engulfment via ELMO1/Dock180, similar to the uptake of apoptotic cells. Subsequently, it has been reported that BAI1 mediates macrophage reactive oxygen species production and microbicidal activity through activation of the Rho family guanosine triphosphatase Rac1 (12). These observations further established BAI1 as a phagocytic receptor of macrophages.

Transcriptome (and proteome) analyses of purified cell populations and, more recently, even single cells is greatly deepening our knowledge about the spatial organization of gene expression. We noticed that omics studies directed at leukocytes consistently detect expression of subfamily E and G adhesion GPCRs, but fail to identify subfamily B receptors, including BAI1 (4, 6, 7). To clarify this discrepancy, we analyzed microarray, CAGE (cap analysis gene expression) and RNA sequencing, and protein mass spectrometry data of primary monocytes, monocytes maturated *in vitro* under stimulating conditions, macrophage cell lines, as well as bone marrow-derived and primary tissue-derived macrophages. We included all types of monocytes/macrophages, in which *Adgrb1*/*ADGRB1* expression has been reported, with the exception of gastric phagocytes (Table 1). Among other data sets, we evaluated adhesion GPCR transcriptomes (20) and proteomes (23) of classical, intermediate, and non-classical monocytes (Figures 1A,B). Moreover, we examined 299 transcriptomes of monocytes activated with 28 different stimuli, including pattern recognition receptor ligands, cytokines, and metabolic cues (19) (Figure 1C). In none of these and numerous other data sets (Table 1), we obtained evidence that monocytes or monocyte-derived macrophages express *Adgrb1*/*ADGRB1*, while known gene expression patterns of subfamily E adhesion GPCRs were fully confirmed (6, 7).

**Table 1:** Studies reporting and studies failing to find expression of *Adgrb1*/*ADGRB1* (BAI1) in monocytes/macrophages.

**Cell type**	**Reporting expression**	**Failing to find expression**
Mouse monocyte/ macrophage cell lines J774A.1 and RAW264.7	RT-PCR, IB (3)	RNAseq (13–15)
Human monocyte/ macrophage cell line THP-1	RT-PCR, IB (9)	RNAseq (16, 17) (http://www.proteinatlas.org)
Monocytes and monocyte-derived macrophages	Microarray (18), IB (9)	Microarray (19), CAGEseq (20), RNAseq (17, 21, 22), MS (23)
Bone marrow-derived macrophages	RT-PCR (11)	RNAseq (14, 24)
Tissue-derived macrophages	RT-PCR, IB (9)	RNAseq (24, 25) (https://www.immgen.org/)
Microglia	IHC (26), ISH (10)	RNAseq (24, 25, 27–32)

*CAGEseq, CAGE sequencing; IB, immunoblot; IHC, immunohistochemistry; ISH, in situ hybridization; MS, mass spectrometry; RNAseq, RNA sequencing; RT-PCR, reverse transcriptase-polymerase chain reaction*.

**Figure 1:**
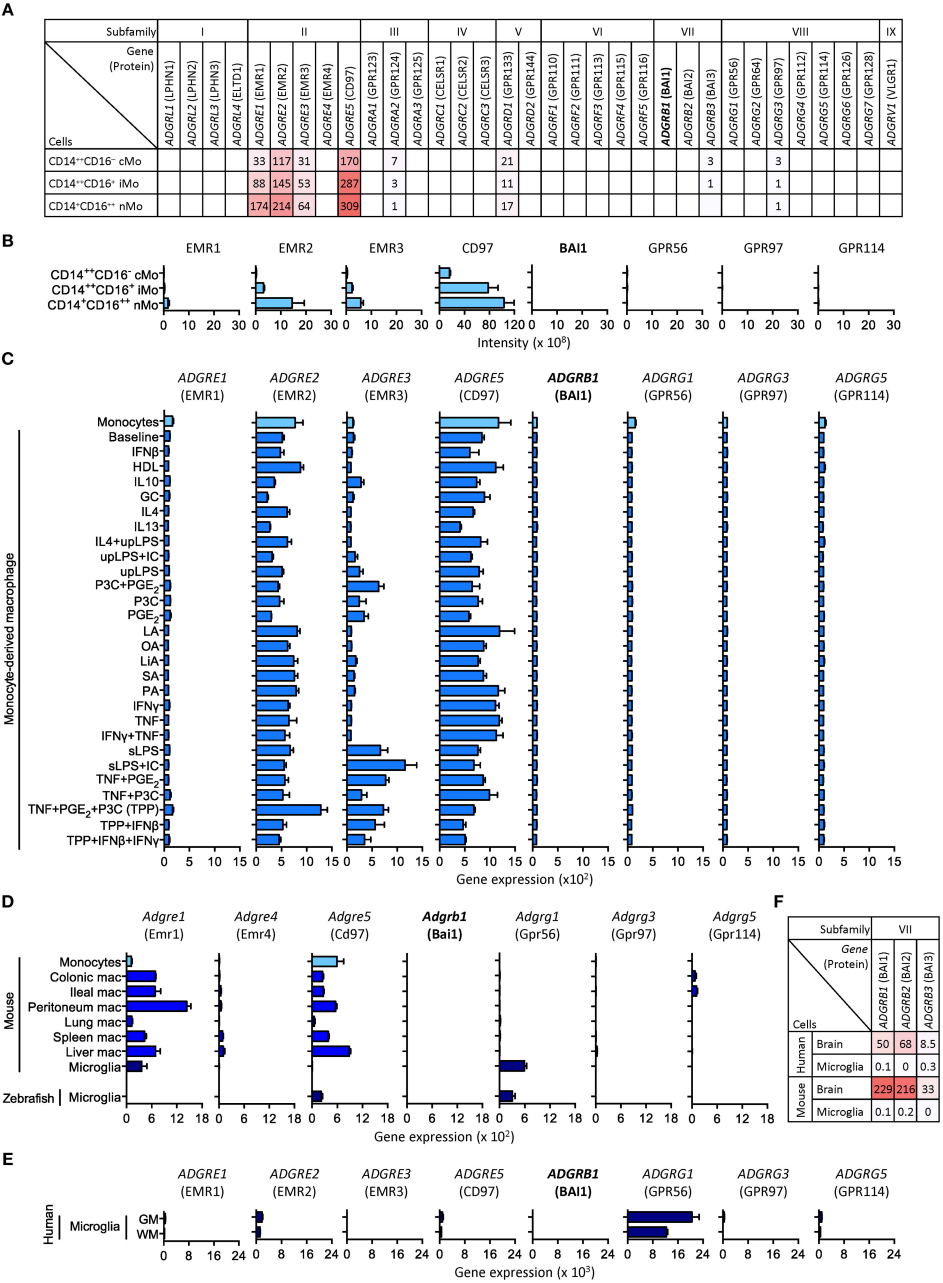
Selected expression profiles of adhesion GPCRs in monocytes, monocyte-derived macrophages, and microglia. **(A)** CAGE sequencing of circulating human monocytes (20). **(B)** Protein mass spectrometry of circulating human monocytes (23). **(C)** Microarray of human monocytes activated with 28 different stimuli (19). **(D)** RNA sequencing of resident mouse macrophages as well as mouse and zebrafish microglia (25, 27). **(E)** RNA sequencing of resident human grey and white matter (GM and WM) microglia (32). **(F)** RNA sequencing of mouse and human brain lysates and microglia (30). Note the consistent lack of BAI1 (*Adgrb1*/*ADGRB1*) expression in all data sets. Expression of EMR1 to EMR4 (*Adgre1*/*ADGRE1* to *Adgre4*/*ADGRE4*) in human and mouse reflect their evolutionary diversification: (i) in contrast to its mouse homolog, F4/80, human EMR1 is weekly expressed by monocytes and macrophages; (ii) mice lack the genes encoding EMR2 and EMR3; (iii) the gene encoding EMR4 has become inactivated in human (33).

Knowledge of genome-wide gene expression in tissue-resident macrophages, so far, is mainly based on studies in mice. In transcriptomes of seven types of macrophages, *Adgrb1* was not detected (25) (Figure 1D). These transcriptomes also included microglia, for which a distinct role for BAI1 in the engulfment of neurons has been described in zebrafish (10). Zebrafish express homologs of most adhesion GPCRs, including BAI1 (34). Yet, by RNA sequencing highly pure microglia from zebrafish, we failed to detect significant levels of *Adgrb1* expression (27) (Figure 1D). Similarly, microglia from mouse and human express *Adgrg1*/*ADGRG1*, but not *Adgrb1*/*ADGRB1* (24, 28–32) (Figures 1D,E).

We also asked whether unusual mRNA properties, e.g., short poly(A) tails, could have hampered the detection of *Adgrb1*/*ADGRB1* transcripts. To exclude this possibility, we included in our comparison RNA sequencing data obtained by reduction of ubiquitously expressed ribosomal (r)RNAs in combination with random primer amplification (13, 14). Moreover, we were able to directly compare sequencing of human microglia RNAs obtained by poly(A) selection and rRNA depletion plus random primer amplification [(32) and Mizee et al., manuscript in preparation], but failed to detect *ADGRB1* transcripts with both methods (data not shown). Furthermore, *Adgrb1*/*ADGRB1* transcripts are found in mouse and human brain lysate (Figure 1F) as well as in mouse neurons, oligodendrocyte progenitors, and astrocytes (28), confirming their detectability.

Our data do not challenge the role of BAI1 as a phagocytic receptor. This biological activity is based on the binding capacity of the N-terminal thrombospondin repeats for “eat-me” signals on apoptotic cells and on the ability of the C-terminal tail to facilitate cytoskeletal rearrangements, and has been proven extensively (3, 11). We question, however, that BAI1 is part of the phagocytic machinery of macrophages. The link with macrophages has been established in primary cells and cell lines overexpressing BAI1 *in vitro*. More recently, Lee at al. investigated the role of BAI1 in the dextran sodium sulfate-induced model of colitis *in vivo*. *Adgrb1*-deficient mice had more pronounced colitis and lower survival, with many uncleared apoptotic cells and inflammatory cytokines within the colonic epithelium. Notably, transgenic overexpression of *Adgrb1* in epithelial, but not in myeloid cells, attenuated colitis severity (35), suggesting that BAI1 mediates clearance of apoptotic corpses within the colonic epithelium. Intestinal epithelial cells may not be the only non-professional phagocytes that engage BAI1. In astrocytes engulfing apoptotic targets, BAI1 showed accumulation within the phagocytic cup (26). Moreover, BAI1 and BAI3 have been described to promote myoblast fusion, a process possibly induced by dying myoblasts (36, 37).

In summary, monocytes and macrophages, including microglia, express the adhesion GPCRs EMR1, EMR2, EMR3, CD97, and GPR56 with different species and cell type specificity. BAI1, an adhesion GPCR with diverse and intriguing functions in angiogenesis, neural development, and apoptotic/microbial engulfment, is hardly expressed by professional phagocytes, and we suggest to reassess the link between BAI1 and macrophage biology.

## Author Contributions

C-CH, MvdP, TvH, and JH generated and analyzed data. C-CH and JH wrote the paper.

### Conflict of Interest Statement

The authors declare that the research was conducted in the absence of any commercial or financial relationships that could be construed as a potential conflict of interest.

## Abbreviations

BAI: brain-specific angiogenesis inhibitor
GAIN: GPCR autoproteolysis-inducing
GPCR: G protein-coupled receptor
GPS: GPCR-proteolysis site.
